# Accelerating European electronic health record exchange format (EEHRxF) implementation across Europe: a policy perspective

**DOI:** 10.3389/fmed.2025.1648170

**Published:** 2025-09-09

**Authors:** Nathan da Silva Carvalho, Alexia Papadimitriou, Robert Stegwee, Hans Gille, Michael Strübin, Christophe Maes, Giorgio Cangioli, Catherine Chronaki

**Affiliations:** ^1^DIGITALEUROPE, Brussels, Belgium; ^2^CEN/TC 251 Health Informatics, Brussels, Belgium; ^3^CGI Netherlands, Amstelveen, Netherlands; ^4^European Health Telematics Association (EHTEL), Brussels, Belgium; ^5^European Institute for Innovation through Health Data (i∼HD), Gent, Belgium; ^6^HL7 Europe, Brussels, Belgium

**Keywords:** interoperability, health data, standards, digital health, regulations and health policy, electronic health records, European electronic health record exchange format

## Abstract

**Background:**

The European Health Data Space Regulation (EHDS), published in the Official Journal since 5 March 2025, requires Electronic Health Record (EHR) systems across the European Union (EU) to adopt harmonized interoperability and logging components using the European EHR Exchange Format (EEHRxF).

**What is at stake:**

The EHDS has come into force and the deadlines for implementation acts are approaching, Clarity on scope, obligations, and implementation options is needed to meet these deadlines.

**Policy options:**

This article examines ways to accelerate EEHRxF adoption, including hybrid implementation models, and coordinated national and EU-level support.

**Recommendations:**

Utilize the European EHRxF Standards and Policy Hub, align national and regional strategies, support SMEs and providers with guidance and funding, and promote digital health literacy to ensure effective EEHRxF implementation.

## 1 Introduction

**FIGURE 1:**
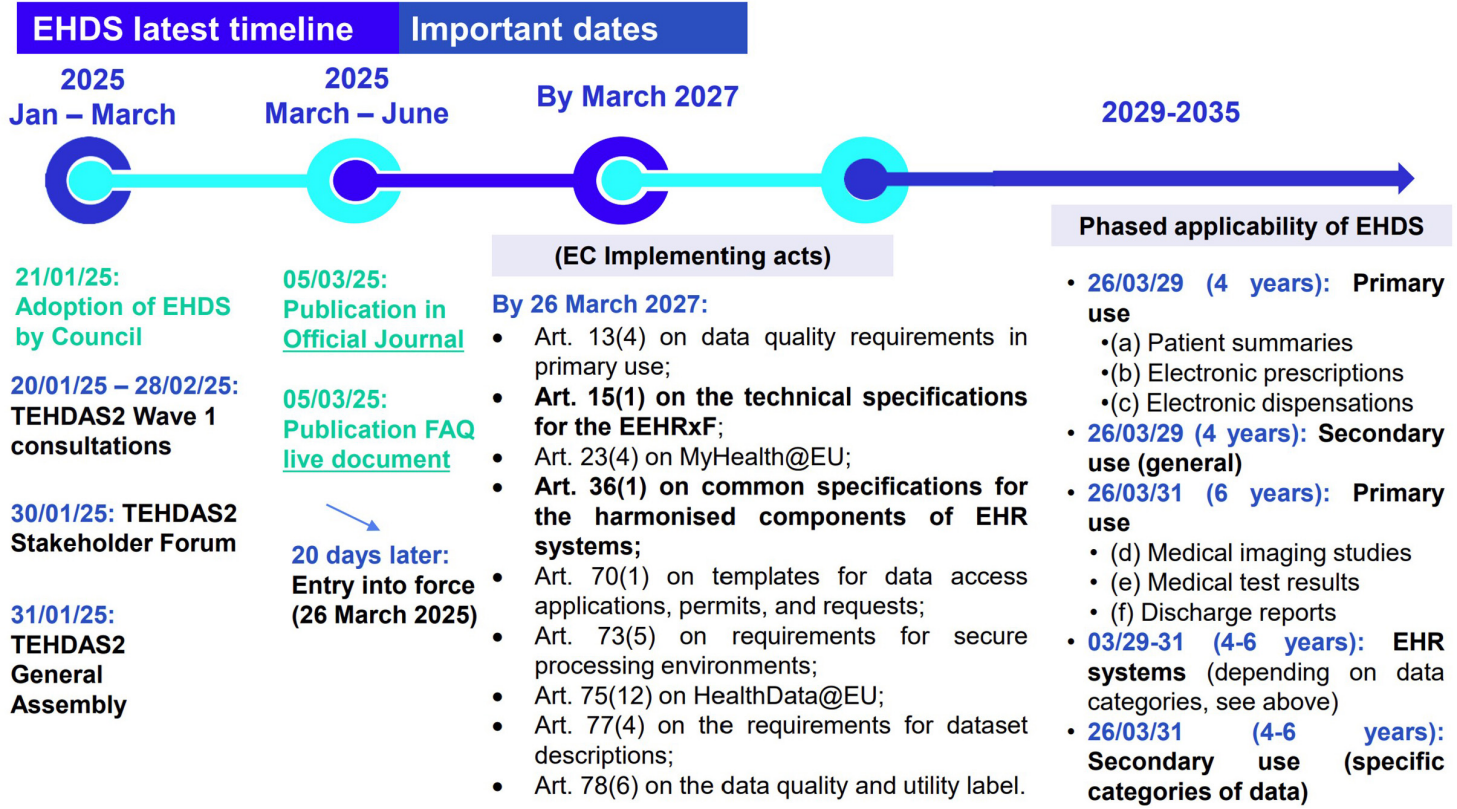
EHDS timeline as from official EHDS publication 5 March 2025.

With the EHDS Regulation now in force (1), the focus shifts from legislation to real-world implementation. Central to this is the European Electronic Health Record Exchange Format (EEHRxF), designed to make health data accessible and interoperable across Member States. With EEHRxF, natural persons across Europe will have the right to access, to see who accessed, to share, to rectify, and to insert data into their EHR (EHDS articles 3–10) (1). Successfully implementing the EEHRxF requires more than regulatory compliance; it demands coordinated action and engagement across the entire health data ecosystem throughout the European Union (EU). To comply with the EHDS Regulation, stakeholders, ranging from healthcare providers and EHR manufacturers to national authorities, will need to follow an implementation timeline (see Figure 1).

The EHDS Regulation entered into force in March 2025, initiating a transition period. As specified in EHDS article 15(1), by 26 March 2027 the European Commission must adopt key implementing acts with the detailed technical specifications for the EEHRxF (2). Starting from March 2029, Member States are required to support the exchange of the first group of priority data categories (patient summaries and ePrescriptions/eDispensations) for primary use, while secondary use rules will also begin to apply. By March 2031, the exchange of the second group of health data categories (medical imaging, lab results, and hospital discharge reports) must be fully operational, alongside the application of secondary use rules for more complex data types, including genomic data (3).

This article aims to identify the key challenges and policy choices to facilitate and accelerate EEHRxF implementation across Europe, drawing on experience from current and past EU- funded projects, European and global initiatives. It offers concrete, actionable recommendations to ensure the EEHRxF is not just a regulatory milestone, but a trusted and widely adopted enabler of digital health across Europe offering citizens access and control to their health data.

This policy brief introduces distinct innovations to EHDS literature by bridging policy, technical feasibility, and stakeholder-driven implementation. While previous studies have primarily focused on regulatory interpretation and theoretical frameworks for interoperability [e.g.,(4, 5)], this brief advance the field of digital health by offering a comparative analysis of four EEHRxF implementation models, introducing a novel voluntary Industry Label for Interoperability, and operationalizing patient empowerment via the “Yellow Button” concept. These contributions reflect practical insights from the xShare project and draw on direct industry engagement with key actors, thereby addressing a critical gap in prior research which lacks applied and market-validated policy tools.

## 2 Challenges, policy options and implications

Meeting the EHDS regulation deadlines presents significant challenges, particularly regarding the scope of EHR systems and the development of robust technical specifications. The following sections outline these challenges and explore related policy options and implications.

To help tackle these challenges, several EU-funded projects, such as xShare, Xt-EHR, and XpanDH, have been launched to support the practical implementation of the EHDS. These initiatives play a role in shaping technical standards, testing environments, and tools that can guide real-world adoption. One example is the xShare project’s “Yellow Button,” a button designed to be placed in electronic health record system and other patient-facing digital health applications that will allow individuals easily access and share their health data in the EEHRxF format. While not a regulatory requirement, the Yellow Button serves as a practical demonstration of how citizens’ data rights under the EHDS could work in everyday settings. Its inclusion here offers a concrete example of how policy goals can be translated into meaningful digital experiences for patients across Europe.

### 2.1 Defining practical EEHRxF specifications

One of the implementation challenges in the EEHRxF is defining clear specifications that can work for the stakeholders when exchanging health data. These specs must enable structured data exchange (e.g., patient summaries, e-prescriptions) within and across Europe’s diverse health systems. Without a stable, well-governed framework, constant changes to specifications risk disrupting the long development cycles of EHR systems, increasing costs and causing delays. These delays will hit smaller manufacturers and less mature Member States hardest, exacerbating digital health inequalities across the EU, an issue previously acknowledged in the European Commission Recommendation (EU) 2019/243 (6).

Stakeholders from projects like X-eHealth, XpanDH, and xShare have stressed the need for clear, harmonized specifications with a predictable lifecycle. The legal mandate is clear: the European Commission must adopt implementing acts by March 2027, based on recommendations from the Xt-EHR Joint Action and ongoing work in other projects. The xShare project has, to achieve its vision of enabling real-time patient access, invested in specifications for the experimental xShare yellow button (7). This button allows citizens to download their health data in clear HL7 FHIR specifications. With change management and alignment built into the process, the specifications in use are co-created with the industry and interoperability competence centers. The xShare yellow button specifications are regularly aligned with the preparatory work on the Implementing Acts spearheaded by Xt-EHR and standards developing organizations like CEN, HL7 Europe, IHE-Europe, CDISC, IEEE, and SNOMED. The xShare yellow button mediates for three core functionalities, namely (I) download/upload, (II) one-time share, (III) link for health data in EEHRxF to flow among applications in a trustworthy manner mediated by patients. The Table 1 below lists the HL7 FHIR specifications currently used as part of the first xShare yellow button implementation guide (7), and references will be updated as new versions of the HL7 FHIR specifications for these categories will be made available.

**TABLE 1:** HL7 FHIR technical specifications, pre-implementing the EEHRxF in the xShare yellow button.

EHDS priority categories	
Patient summaries	HL7 International patient summary (v1.1.0)
Electronic prescriptions	HL7 Europe medication prescription and dispense (v 0.1.0-ballot)
Electronic dispensations	HL7 Europe medication prescription and dispense (v 0.1.0-ballot)
Medical imaging studies and related imaging reports	HL7 Europe medication prescription and dispense (v 0.1.0-ballot)
Medical test results, including laboratory and other diagnostic results and related reports	HL7 Europe laboratory report (v 0.1.1)
Discharge reports	HL7 Europe hospital discharge report (v 0.1.0-ballot)

### 2.2 Addressing industry barriers in EEHRxF adoption and the scope of EHR systems

**FIGURE 2:**
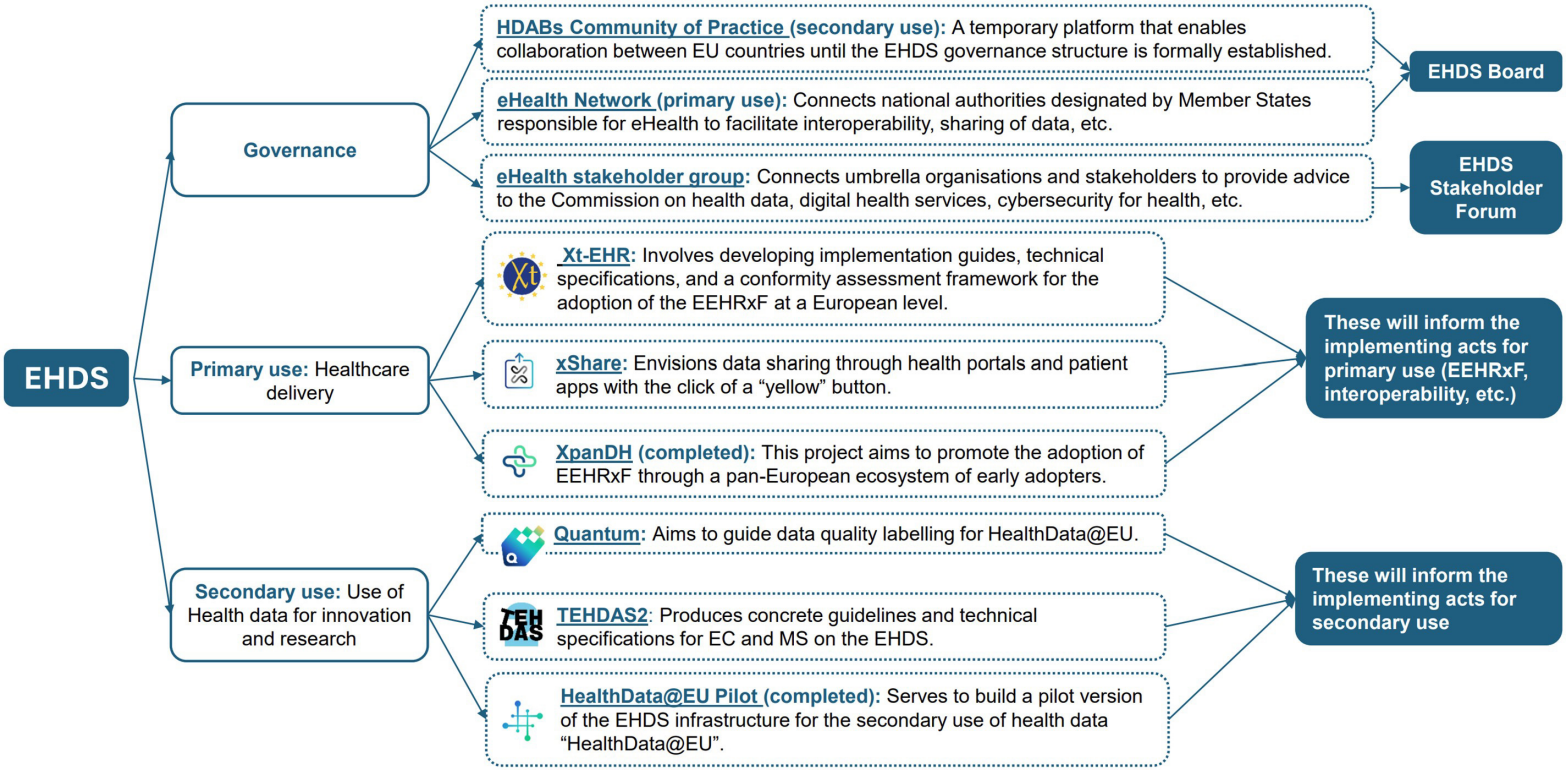
EHDS supporting initiatives (not exhaustive view).

The development of the EEHRxF is shaped by a wide range of EU projects, standardization efforts, and working groups (Figure 2). While this shows strong commitment from the digital health community to facilitate EEHRxF adoption, those parallel developments also create challenges: timelines are fragmented, governance is unclear, and manufacturers struggle to stay involved in all initiatives due to limited resources. This fragmentation makes it difficult for stakeholders to follow or contribute effectively.

Furthermore, EHDS article 30(1) mandates compliance with the EEHRxF specifications for EHR systems. The definition of “EHR systems” in the regulation is broad and could encompass any connected device, EHDS, article 2(2k) (1) “*EHR System means any system whereby the software, or a combination of the hardware and the software of that system, allows personal electronic health data that belong to the priority categories of personal electronic health data established under this Regulation to be stored, intermediated, exported, imported, converted, edited or viewed, and intended by the manufacturer to be used by healthcare providers when providing patient care or by patients when accessing their electronic health data*.” Digital health infrastructures in European hospitals include many connected systems including some highly specialized technologies, many of which could now fall within its scope. As a result, there is considerable uncertainty among implementers about which systems need to comply with EHDS. The wait for stable specifications until 2027, the ambitious timeline requiring compliance by 2029, and the long innovation and development cycles of advanced medical technologies: these factors present a risk.

The European EHRxF Standards and Policy Hub (also known just as “the Hub”), created and incubated under the xShare project, is designed to tackle the issue of EEHRxF fragmentation with the aspiration to become the single point of reference for information on implementing or using the EEHRxF getting ready for the EHDS. As a neutral space, the Hub connects public authorities, industry, standards development organizations, and associations of academics, healthcare professionals and healthcare providers, patients and other stakeholders. The Hub will help to ensure that technical guidance, implementation efforts, and projects are aligned with the EHDS regulation and the EEHRxF.

The Hub’s primary function is to support EHR system developers (manufacturers) and implementers (such as healthcare provider and patient service organizations) in identifying and applying the right technical resources to achieve system interoperability at all levels of the Refined eHealth European Interoperability Framework (8). In this regard, the following key resources are made available:

1. **The EEHRxF website** (9): Provides an overview of the EEHRxF in the context of the EHDS (EEHRxF in a Nutshell), guidance to users of the EEHRxF, and an overview of the work being done on the EEHRxF.
2. **The business use case repository** (10): Which documents different business use cases for which the EEHRxF is being used? The repository serves both as a knowledge base and as an advocacy tool to demonstrate the relevance and impact of the EEHRxF in supporting the EHDS and broader digital health transformation goals.
3. **The X-Bundle registry** (11): A compilation of references to all relevant specifications, tools and supporting materials from a variety of sources. These assets can be organized in different ways, focusing on the health information domains, supporting specific business use cases, addressing specific levels of the Refined eHealth European Interoperability Framework (8).
4. **Adoption kits for stakeholders** (12): Tailored toolkits to support the adoption and implementation of the EEHRxF among key user groups, citizens, healthcare professionals, procurers, and developers. These kits will include step-by-step guidance, training resources, communication templates, and best practices to ease integration and foster trust in EEHRxF-based systems.
5. **Community of excellence for interoperability leaders** (13): An active network bringing together manufacturers, standards experts, and other stakeholders to share experiences, co-develop guidance, and align on good practices. This community serves as a collaborative forum to address challenges, incubate innovations, and build consensus on evolving standards for the EEHRxF.

### 2.3 Policy options: implementing the harmonized software components for EHR systems

A key challenge in operationalizing the EHDS lies in how to implement the access to and sharing of electronic personal health data, such as foreseen for the xShare yellow button. One way to approach this is through the harmonized software components of EHR systems: “*EHR systems shall include a European interoperability software component for EHR systems and a European logging software component for EHR systems (the ‘harmonized software components of EHR systems’)*” EHDS, article 25(1) (1). Another way is through the electronic health data access services mentioned in EHDS Article 4(1): “*Member States shall ensure that one or more electronic health data access services at national, regional or local level are established, thereby enabling natural persons to access their personal electronic health data and exercise their rights provided for in Articles 3 and 5 to 10.*”

These two different ways in which to implement access and sharing, have led to three implementation models emerging from ongoing policy and project discussions, as shown in Table 2. These models have clear advantages and limitations. A decentralized approach encourages long-term system-wide integration and patient empowerment at the point of care but could pose major financial and technical challenges for under-resourced or fragmented systems. A centralized model, by contrast, may reduce the implementation burden at the local level but risks disconnecting patients from their point-of-care providers and creating a bottleneck for interoperability. Finally, a hybrid model requires coordination between decentralized and centralized models, ideally via the European EHRxF Standards and Policy Hub, which would harmonize standards and facilitate stakeholder dialogue, ensuring consistent implementation and local flexibility. The Hub acts as a neutral ground, aligning technical standards, sharing best practices, and facilitating communication between national authorities, healthcare providers, and technology vendors, ensuring interoperability while accommodating local contexts.

**TABLE 2:** Implementing access to electronic personal health data.

Implementation model	Advantages	Implications	Stakeholders impacted
Option 1: Decentralized. Local EHR portals implement EEHRxF directly. Each healthcare provider (e.g., hospital, clinic) ensures that their EHR system includes the interoperability and logging components required by Article 25. Patients access their health data and exercise their EHDS rights directly through provider-level portals.	Gives patients direct access to the point of care and encourages wider, system-level integration. It also allows for local flexibility and innovation.	This places a high burden on individual providers, particularly in less digitally mature settings, and may lead to uneven implementation across regions, confusion for European citizens and lack of interoperability. Regular testing and certification are necessary.	Healthcare providers, patients, EHR system vendors, regulatory authorities
Option 2: Centralized. Member States provide a single platform. The national or regional health authority implements a central portal that interfaces with local EHR systems. Patients access, download, and share their EEHRxF-compliant data through this centralized service.	Easier to manage in countries with fragmented systems or fewer digital resources. Creates a more consistent national experience for patients.	Can slow down adoption at the provider level to fit the one-size-fits-all approach and risks creating dependence on national timelines and infrastructure. Natural persons may have access to a limited part of their health data.	National health authorities, patients, healthcare providers
Option 3: Hybrid approach. Mixed model depending on context. Combines centralized national services with decentralized provider-level implementations. Suitable for countries with regionalized healthcare systems or varying provider readiness.	Offers flexibility and stepwise implementation while allowing countries to scale gradually based on local readiness. A good balance between control and customization.	Needs strong coordination to ensure consistency. Without it, patient experience and technical standards may vary widely. Regular testing and certification are necessary.	National and regional health authorities, healthcare providers, patients

For many Member States, particularly larger or federal ones, a hybrid approach may prove more realistic. Some regions or providers may adopt direct implementation models, while others rely on national infrastructure. This flexibility is important but must still ensure full compliance with the rights enshrined in EHDS Articles 3–10, including access to priority data categories in the EEHRxF format and the ability to share them securely. Each Member State has a different starting point in terms of digital maturity, resulting in probable variance in their approach. Continuous alignment or a joint effort with various stakeholders, e.g., via the Hub, is recommended, to enable this range of approaches while safeguarding consistency and interoperability. In short, implementing access to personal electronic health data, including the use of the harmonized components of EHR systems, as mandated by the EHDS is a complex task that requires aligning legal rights, institutional models, governance structures, and real-world infrastructure to ensure that all Europeans, regardless of where they live or receive care, can benefit from a connected digital health ecosystem.

## 3 Actionable recommendations

**(a) Deliver regulatory guidance regarding the scope of the regulation:** The EHDS presents a considerable opportunity for innovators and manufacturers of medical technologies and systems, however there is a risk that regulatory uncertainty and strict timelines create difficulties. More guidance is needed about the scope of the regulation, developed in consultation with experts from vendors and users, incorporating feedback from early adopters, to ensure the guidance considers the complexity and diversity of connected medical technologies.

**(b) Align national strategies and communication around EEHRxF scope:** To support smoother implementation of the EHDS Regulation, Member States should avoid introducing additional regulatory or technical specifications beyond what is already set out in the regulation and its upcoming implementing acts. Clearly, as stated in EHDS article 42(2) (1), “*national requirements or provisions [.] shall not adversely affect the harmonized software components of EHR systems*”. Consistent communication about the scope of EEHRxF is key to ensuring clarity for all stakeholders and to fostering a more interoperable health data environment across Europe. At the same time, it is important to acknowledge that some variation is inevitable regarding the national implementation strategy for harmonized software components and the electronic health data access service for individuals. While manufacturers can meet EHDS specifications by self-declaring compliance through testing in any approved national digital environment, certain Member States may still require additional testing to meet local rules. To keep adoption efficient and avoid unnecessary delays to market entry, national testing environments should follow European Commission guidance as closely as possible and make any extra specifications clear and predictable for manufacturers. Engaging the Hub to orchestrate mutual alignment in close collaboration with the broader stakeholder community is recommended.

**(c) Consider a critical role for the Hub in the upcoming EEHRxF Support Center:** Deliver clarity that the Hub, as a central point of contact for the EEHRxF, can streamline stakeholder outreach, minimize duplication across projects, ensure that evolving technical specifications align with the EHDS regulation, and link the EEHRxF to global harmonization efforts. As such, the Hub is clearly positioned to support activities of the forthcoming EEHRxF Support Center (14), especially considering that xShare has established a legal entity to ensure sustainability for the Hub beyond the end of the xShare project. This legal entity – the European Standards for Health Interoperability Alliance (ESHIA) – is being formed in 2025 as an international non-profit association, backed by Europe’s leading standards development organizations, stakeholder groups, and innovation centers and with the stated mission to support the EHDS by operating the Hub. In conformity with Belgian law, the eighteen founding members have elected a Board of ten and an inaugural executive committee of three. Its revenues will include membership dues, income from interoperability assets, and fees for services. When the xShare project ends its financial support of the Hub in November 2026, ESHIA will inherit the Hub and its assets and ensure the Hub’s continued role in a dynamically evolving environment.” (15).

**(d) Assess the xShare Yellow Button specifications (HL7 FHIR Implementation Guides) as a viable option to prepare the industry for European-wide EHDS readiness:** EEHRxF technical specifications should align with international standards to the greatest possible extent. These may include elements such as defined profiles, machine-readable validation rules, and illustrative real-world examples. The xShare yellow button enables natural persons to share their health data in HL7 FHIR. The technical specifications for the xShare yellow button prepare the ground for demonstrating conformance with the EEHRxF through close collaboration with the Xt-EHR project. Thus, they serve as a helpful reference and implemented proof of concept when drafting the EEHRxF technical specifications in Xt-EHR that will provide input to the implementing acts planned to be released by the European Commission in March 2027.

**(e) Structure stakeholder engagement based on product classes and use cases:** Instead of relying on broad, one-size-fits-all stakeholder groups, future engagement should be tailored to the actual use of specific types of digital health products defined in the EHDS Regulation, such as EHR systems, medical devices, wellness apps, and high-risk AI applications. This focused approach would allow the European Commission to better address the unique challenges and needs of each category. For example, in the case of EHR systems, the Xt-EHR project provides further classification of EHR systems and useful guidance for classifying different types and their relevance to the EEHRxF technical specifications. All integration needs to be grounded in real-world use cases, so these engagements may help ensure that the EEHRxF elements are clearly understood and consistently applied across the diverse digital health landscape.

**(f) Support manufacturers and healthcare providers with contextual guidance and funding:** National funding should focus on strengthening healthcare provider infrastructure and improving digital literacy among professionals and patients to support EEHRxF adoption. At the EU level, funding, especially for SMEs, can help manufacturers meet interoperability specifications, reflecting the single market goals of the EHDS. Examples such as the EU4Health funding program (e.g., the €12.8 million tender for diagnostic devices) and national toolkits like Catalonia’s TIC Salut Social provide practical models for supporting digital adoption and capacity-building. National toolkits and training, alongside support from the Hub, can further guide implementation efforts across an educated ecosystem. Overall, the plan needs to multiply drivers/incentives (administrative simplification, process orchestration, financial meaningful use incentives etc.).

**(g) Use the testing experience of xShare for the digital testing environment to be made available by the European Commission:** In setting up the digital testing environment foreseen in Article 40 of the EHDS regulation, article 40 (1), the European Commission should consider best practices, use cases, and project outcomes from xShare. Feedback and lessons learned from preliminary EEHRxF testing made in the context of the xShare Industry Label using the IHE Europe Gazelle testing suite (16) and national programs can support the drafting of the implementing acts and the development of the future digital testing environment to be made available by the European Commission.

## 4 Conclusion

With the EHDS now in force, the focus has shifted to putting its vision into practice. The EEHRxF is at the heart of that effort, enabling patients to access and share their health data across borders and systems.

With EHDS implementation deadlines fast approaching, a clear and coordinated response is essential. Member States must recognize the role of the Hub and actively engage with it by 2026 to ensure alignment on interoperability, certification, and implementation practices. Doing so will help avoid fragmentation, support timely compliance with Article 25 of the EHDS Regulation, and ensure that patients across Europe can consistently access and share their health data. Robust national strategies, informed by the Hub’s guidance, will be key to achieving a connected and patient-centered EHDS.

Successful adoption of the EHDS and the EEHRxF will rely heavily on the handholds offered to both policy makers as well as industry players and healthcare professionals. A potential delay of implementation or a lack of coordination is likely to lead to a loss of momentum and will jeopardize Europe’s ambitions to work toward a single market for digital health services and improved patient care.

This policy brief has highlighted the main roadblocks and offered practical ways to move forward. What is needed now is real collaboration across governments, healthcare providers, industry, and the wider digital health community to move toward actual implementation. No one can deliver this alone. By aligning efforts, investing in the right tools, and supporting both providers and vendors, Europe can make secure, seamless health data exchange a reality, and ensure that the EHDS delivers meaningful benefits to citizens across the EU.
